# SARS-CoV-2-Associated Guillain-Barre Syndrome Obscured by Diabetes Mellitus Peripheral Neuropathy

**DOI:** 10.7759/cureus.14209

**Published:** 2021-03-31

**Authors:** Miranda Brown, Alana Petrassi, Britta L Bureau, Nabeel Khan, Pinky Jha

**Affiliations:** 1 Internal Medicine, Medical College of Wisconsin, Milwaukee, USA; 2 Internal Medicine/Neurology, Medical College of Wisconsin, Milwaukee, USA

**Keywords:** guillain-barre syndrome, covid-19, sars-cov-2, diabetes

## Abstract

Multiple neurological complications, including Guillain-Barre syndrome (GBS), have been reported in association with the novel severe acute respiratory syndrome coronavirus 2 (SARS-CoV-2) outbreak. GBS has well-known associations with viruses such as influenza, human immunodeficiency virus, Zika, severe acute respiratory syndrome, Middle East respiratory syndrome, Epstein-Barr virus, and cytomegalovirus. Till date, there have been around 50 distinct published cases of GBS occurring concurrently or shortly after SARS-CoV-2 infection. This report describes the case of a 53-year-old male who presented with bilateral extremity paresthesias two weeks after a positive SARS-CoV-2 test. His symptoms were originally thought to be due to underlying diabetic peripheral neuropathy, but as they progressed, he was eventually diagnosed with SARS-CoV-2-associated GBS. Though GBS may not be a common sequelae of SARS-CoV-2 infection, the prevalence of diabetes mellitus-associated peripheral neuropathy is high enough to warrant awareness and prompt recognition of neurological symptoms that deviate from the baseline in individuals with recent, confirmed SARS-CoV-2 infection.

## Introduction

There is an ever-expanding spectrum of documented neurological complications following infection with the novel coronavirus. Along with changes in mental status, intracranial infection, and increased incidence of cerebrovascular accidents, severe acute respiratory syndrome coronavirus 2 (SARS-CoV-2) infection has been associated with the development of Guillain-Barre syndrome (GBS) [1]. This association was first documented early in the pandemic in a correspondence in the New England Journal of Medicine submitted by Italian physicians who had witnessed the development of the syndrome in five patients from February to March of 2020 [2]. Since then, the development of GBS in patients with a recent, confirmed case of SARS-CoV-2 infection has been documented by clinicians and researchers around the globe.

GBS is an acute immune-mediated polyradiculopathy thought to be triggered by an acute infection. GBS has well-known associations with viruses such as influenza, human immunodeficiency virus, Zika, severe acute respiratory syndrome, Middle East respiratory syndrome, Epstein-Barr virus, and cytomegalovirus. The main pathological mechanism is thought to be the production of autoantibodies during the immune response to a pathogen which then cross-reacts with peripheral nerve components via sharing of cross-reactive epitopes, a phenomenon called molecular mimicry. In SARS-CoV-2 infection, though there is a correlation, the exact mechanism of immune-mediated nerve damage has not been confirmed, and production of anti-ganglioside antibodies has not been verified [3].

The classic clinical manifestation includes a symmetric ascending muscle weakness with absent or depressed deep tendon reflexes; however, it is a heterogeneous syndrome with multiple variants. One of the most common variants is acute inflammatory demyelinating polyradiculopathy (AIDP), which results from the demyelination of both motor and sensory nerves and presents with ascending paresthesias and weakness [4]. In a meta-analysis of 50 cases of SARS-CoV-2-associated GBS documented in 37 different papers, 66% (n = 33) of cases presented as AIDP [4]. GBS typically progresses over two weeks, peaks within four weeks, and then patients slowly regain function over weeks to months [5]. The diagnosis is often made clinically, although electrodiagnostic studies such as nerve conduction studies or electromyography can aid in making a diagnosis. Treatment typically consists of a combination of intravenous immunoglobulins (IVIG) and/or plasmapheresis, which is most effective if started early in the disease course [5].

## Case presentation

A 53-year-old Middle Eastern male with a history of type II diabetes complicated by peripheral neuropathy presented to the emergency department with numbness and tingling in hands and feet bilaterally nine days after a positive SARS-CoV-2 test. He reported that his paresthesias were different in distribution from his baseline. The patient was discharged with a referral to neurology. The following day he presented with paresthesias extending to the groin and bilateral face. Paresthesias were attributed to acute worsening of his chronic diabetic neuropathy with overlying hyperventilation due to anxiety, and he was admitted for observation.

On day one of admission, vitamin B12, folate, methyl-malonic acid, serum protein electrophoresis, and urine protein electrophoresis were within normal limits and SARS-CoV-2 IgG antibodies were reactive. Computed tomography of the spine revealed no acute fracture or deformity. On day three, neurology was consulted, and his examination did not demonstrate any focal weakness. On day four, his examination was significant for left-sided facial droop, dysarthria, bilateral grip weakness worse on the left, right foot drop, and absent bilateral biceps and patellar reflexes. At this point, continued, rapid progression of peripheral neuropathy resulted in the suspicion of GBS and initiation of IVIG 0.4/kg/day for four days. On day five, the patient required supplemental oxygen, and on day seven, he had increasing oxygen requirements due to progressive ascending paralysis involving respiratory and bulbar muscles. On day nine, the patient was placed on bilevel positive airway pressure due to respiratory distress and was transferred to the medical intensive care unit. On day 10, he was intubated due to diaphragmatic weakness. Five doses of plasmapheresis were given on day 17 through day 22. His oxygen demands improved, and he was extubated on day 22. The patient received two additional doses of IVIG on day 23 and 24 with improvement in symptoms. Nerve conduction studies on day 23 showed large-fiber sensorimotor polyneuropathy with findings of axonal demyelination, consistent with the clinical suspicion of GBS. The patient continued to improve clinically after treatment and was discharged to a long-term rehabilitation center.

## Discussion

Neurological manifestations have been reported in approximately 36.4% of the patients infected with SARS-CoV-2 [1]. Based on our literature review in March 2021, this is the 51st case report of GBS following a SARS-CoV-2 infection. This case and course of illness is consistent with the previous case reports describing GBS, specifically the AIDP subtype, after a SARS-CoV-2 infection. This patient was a male in his 50s, which is the median age of other cases [6]. In addition, the patient developed rapidly progressive ascending paralysis nine days after testing positive for SARS-CoV-2; similarly, previous cases have reported a mean time to neurological symptoms as 11 days plus or minus five days [6]. The patient also responded to the standard treatment for GBS of a combination of IVIG 0.4 mg/kg/day and plasmapheresis.

A unique aspect of this case is that the patient’s presenting symptoms were obscured by his underlying diabetic peripheral neuropathy. When the patient presented with complaints of bilateral lower extremity paresthesias, this was initially attributed to an acute worsening of his diabetic peripheral neuropathy, even though the patient stated more than once that his symptoms were not consistent with his prior peripheral neuropathy. This attribution delayed the initial neurology consultation as well as the evaluation and initiation of treatment for GBS.

The prevalence of diabetes in the United States was estimated at 10.5% in 2018 according to the Centers for Disease Control and Prevention [7], with an estimated 50% of the patients having some degree of peripheral neuropathy [8]. Given the prevalence of diabetic neuropathy and the relative immune suppression common in those who have diabetes mellitus, it is likely that cases of AIDP-type GBS secondary to SARS-CoV-2 infection have already and will continue to be misdiagnosed or underdiagnosed. Additionally, a recent study published in Diabetic Research and Clinical Practice found that four diabetic patients admitted to a Barcelona hospital for severe SARS-CoV-2 pneumonia reported new-onset, lower extremity sensory neuropathy following the infection, indicating that serious SARS-CoV-2 infections may provoke a widespread sensory neuropathy in patients with diabetes [9]. In contrast to the case discussed in this report, these four patients did not experience rapid progression of their neuropathic symptoms and continued to exhibit normal deep tendon reflexes. This study complicates an already complex diagnostic problem. Diabetic polyneuropathy can mask the early presentation of SARS-CoV-2-associated GBS, but it appears from this limited sample of patients that serious infection with the virus may also induce new-onset neuropathy in diabetic patients. In view of all of this information, even though SARS-CoV-2-associated GBS appears to be a rare complication and new-onset, infection-induced neuropathy appears to be a possibility in patients with diabetes, the potential life-threatening consequences of GBS make it imperative that physicians caring for diabetic patients with an acute or recent SARS-CoV-2 infection remain vigilant for changes in baseline sensation and motor function. Moving forward, more research is needed to better understand the presentation of GBS, especially in patients with underlying diabetic peripheral neuropathy.

## Conclusions

It remains unclear which patient factors are associated with an increased risk of neurologic complications due to SARS-CoV-2 or by what mechanism the virus induces damage to the peripheral axons, as seen in GBS. Though SARS-CoV-2 has been known to cause an acute inflammatory response, the presence of anti-ganglioside or an autoimmune reaction has yet to be demonstrated. Further research needs to be conducted to discern the exact mechanism for SARS-CoV-2-induced GBS.

We present this unique case of SARS-CoV-2-associated GBS overlying chronic diabetic peripheral neuropathy to add to the literature addressing the neurological complications of SARS-CoV-2 infection, particularly in those with underlying comorbidities. We hope that this case report prompts more research not only into the immunological cause of post SARS-CoV-2 GBS but the patient factors that are associated with it. Additionally, we hope that this case report encourages practitioners to have a heightened suspicion of GBS in their diabetic patients with reported changes in baseline neuropathy after SARS-CoV-2 infection to achieve prompt diagnosis and treatment.
